# Intercellular adhesion molecule 4 and ischemic stroke: a two-sample Mendelian randomization study

**DOI:** 10.1186/s12959-023-00485-4

**Published:** 2023-04-11

**Authors:** Lulu Sun, Daoxia Guo, Yiming Jia, Mengyao Shi, Pinni Yang, Yu Wang, Fanghua Liu, Zhengbao Zhu, Jin Zheng

**Affiliations:** 1 Department of Epidemiology, School of Public Health, Jiangsu Key Laboratory of Preventive and Translational Medicine for Geriatric Diseases, Suzhou Medical College of Soochow University, 199 Renai Road, Industrial Park District, 215123 Suzhou, Jiangsu Province China; 2 School of Nursing, Medical College of Soochow University, Suzhou, China; 3 Department of Neurology, Minhang Hospital, Fudan University, Shanghai, China

**Keywords:** Ischemic stroke, Intercellular adhesion molecule 4, Risk, Mendelian randomization

## Abstract

**Background:**

Experimental studies suggested that intercellular adhesion molecule 4 (ICAM-4) might be implicated in ischemic stroke, but the population-based evidence on the relationship between ICAM-4 and ischemic stroke were limited. Herein, we performed a two-sample Mendelian randomization (MR) analysis to investigate the associations of genetically determined plasma ICAM-4 with the risks of ischemic stroke and its subtypes.

**Methods:**

A total of 11 single-nucleotide polymorphisms associated with ICAM-4 were selected as instrumental variables based on the genome-wide association studies (GWAS) with 3,301 European individuals. Summary-level data about ischemic stroke and its subtypes were obtained from the Multi-ancestry GWAS launched by the International Stroke Genetics Consortium. We used the inverse-variance weighted method followed by a series of sensitivity analyses to evaluate the associations of genetically determined ICAM-4 with the risks of ischemic stroke and its subtypes.

**Results:**

Genetically determined higher ICAM-4 levels were significantly associated with increased risks of ischemic stroke (in the IVW method fitted to multiplicative random effects model: odds ratio [OR] per standard deviation [SD] increase, 1.04; 95% confidence interval [CI], 1.01–1.07; *P* = 0.006; in the IVW analysis with fixed effects model: OR per SD increase, 1.04; 95% CI, 1.01–1.07; *P* = 0.003) and cardioembolic stroke (in multiplicative random effects model: OR per SD increase, 1.08; 95% CI, 1.02–1.14; *P* = 0.004; in fixed effects model: OR per SD increase, 1.08; 95% CI, 1.03–1.13; *P* = 0.003). There was no association of ICAM-4 with the risks of large artery stroke and small vessel stroke. MR-Egger regression showed no directional pleiotropy for all associations, and the sensitivity analyses with different MR methods further confirmed these findings.

**Conclusions:**

We found positive associations of genetically determined plasma ICAM-4 with the risks of ischemic stroke and cardioembolic stroke. Future studies are needed to explore the detailed mechanism and investigate the targeting effect of ICAM-4 on ischemic stroke.

**Supplementary Information:**

The online version contains supplementary material available at 10.1186/s12959-023-00485-4.

## Background

Stroke is the leading cause of death and severe disability around the world [1]. In 2019, there were 101 million stroke cases globally, of which ischemic stroke was 77.19 million and accounted for more than 70%.^1^ Smoking, excessive alcohol consumption, obesity, hypertension, hyperlipidemia, and diabetes are well-established risk factors for ischemic stroke [1, 2]. Although interventions against these traditional risk factors have obtained substantial progress, ischemic stroke is still a major public health problem worldwide [1, 2]. Therefore, further studies are needed to identify novel biomarkers for the prevention and treatment of ischemic stroke.

Intercellular adhesion molecule 4 (ICAM-4), also known as the Landsteiner-Wiener blood group glycoprotein, was a member of the ICAM family expressed primarily on erythrocytes and erythroid precursor cells [3–5]. Compared to other members of ICAMs, ICAM-4 could bind various integrins (e.g., CD11a/CD18, CD11b/CD18, α_4_β_1_, αI_Ib_β_3_, and α_v_ integrins) on cell surface [3, 5]. Previous studies suggested that the interaction of ICAM-4 with integrins played a critical role in cell adhesion, hemostasis and thrombosis [6–8]. In addition, ICAM-4 was reported to mediate the abnormal adhesion of sickle cell to endothelial cells, and then induced platelet-erythrocyte aggregation and blocked blood flow [9]. Platelet-erythrocyte aggregation and endothelial cell dysfunction were well known implicated in the thrombosis, [6–8, 10] so we hypothesized that ICAM-4 played a key role in ischemic stroke etiology. However, to date, there is limited population-based research on the effect of ICAM-4 in the risk of ischemic stroke.

Mendelian randomization (MR) is an emerging epidemiologic method in which genetic variants associated with the exposure of interest are served as instrumental variables to assess the causal effect of a lifelong exposure on diseases [11, 12]. MR design had previously been used to assess the effects of circulating cytokines, serum bilirubin, lipoprotein lipid, and apolipoproteins on the risk of ischemic stroke [13–15]. Besides, two-sample MR design could greatly increase the scope of MR analysis with advantages of two independent samples [16]. Hence, we explored the associations of genetically determined ICAM-4 levels and the risks of ischemic stroke and its subtypes (cardioembolic stroke [CES], large artery stroke [LAS], and small vessel stroke [SVS]) via a two-sample MR study.

## Methods

### Study design

**Fig. 1:**
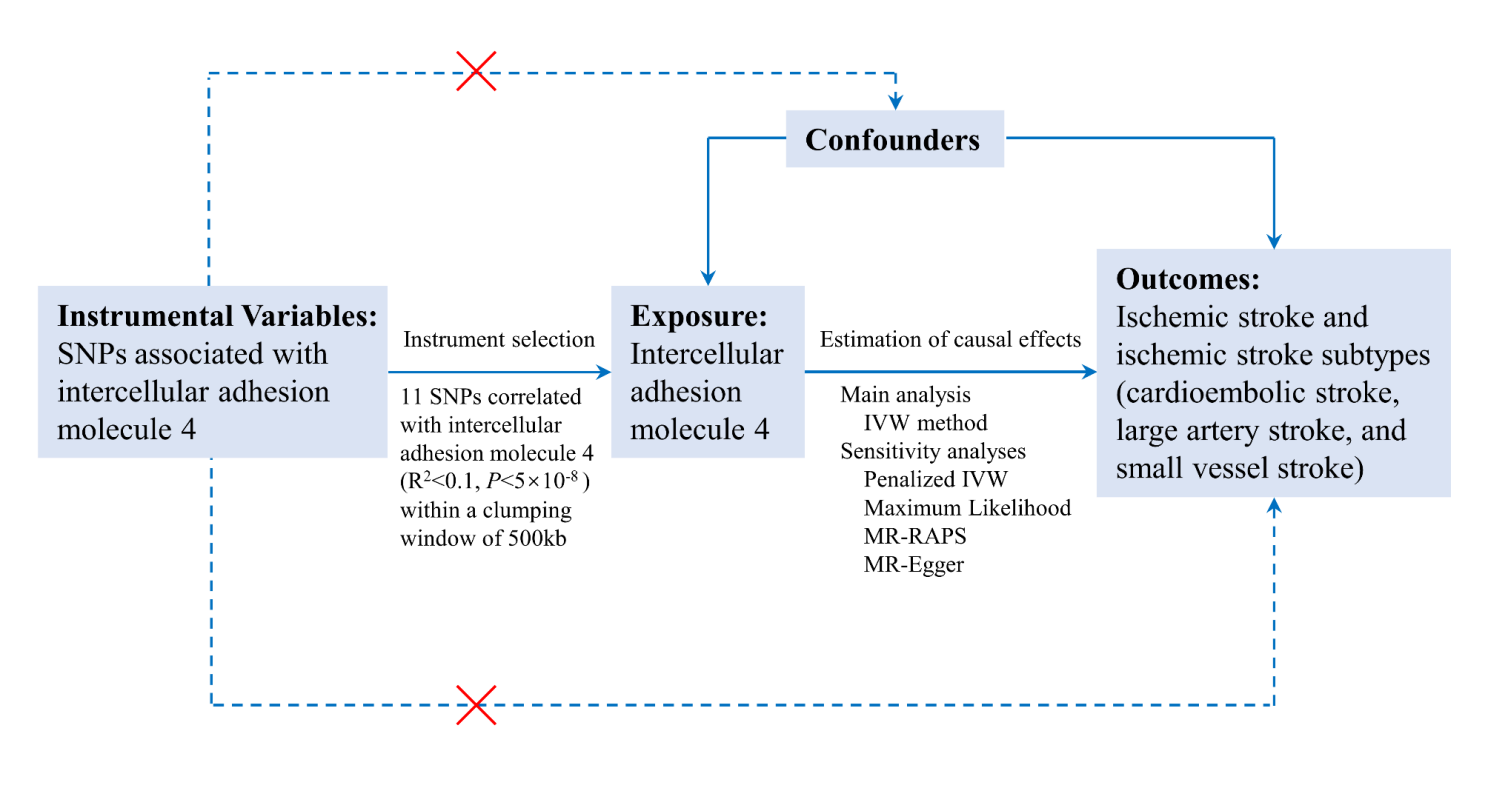
**An overview of the present mendelian randomization study design**. The assumption is that (1) instrumental variables are associated with intercellular adhesion molecule 4 levels, (2) instrumental variables are not associated with confounders, (3) and instrumental variables affect ischemic stroke or its subtypes only through the effects on intercellular adhesion molecule 4 levels Abbreviations: SNP, single-nucleotide polymorphism; IVW, inverse-variance weighted; MR-RAPS, mendelian randomization robust adjusted profile score

The present study was reported using the Strengthening the Reporting of Observational Studies in Epidemiology using Mendelian Randomization (STROBE-MR) guideline [17]. As shown in Fig. 1, we designed a two-sample MR study to systematically investigate the associations of genetically determined ICAM-4 levels and the risks of ischemic stroke and its subtypes. We selected single-nucleotide polymorphisms (SNPs) that achieved genome-wide significance (*P* < 5.0 × 10^− 8^) for the ICAM-4 levels identified by Sun et al. as instrumental variables in the MR analysis [18]. The summary genetic data about ischemic stroke and its subtypes were obtained from the Multi-ancestry Genome-Wide Association Study launched by the International Stroke Genetics Consortium (MEGASTROKE) [19]. All participants in the present MR analysis were subjects of European ancestry. The protocol and data were approved by the ethics committee of the original genome-wide association studies (GWASs), and written informed consent was obtained from each participant prior to data collection.

### Instrumental variables for ICAM-4

The summary genetic data about ICAM-4 were derived from a genomic atlas analysis of the human plasma proteome conducted by Sun et al. [18] They randomly selected two non-overlapping sub-cohorts from INTERVAL study, which comprised about 50,000 participants nested within a randomized trial of varying blood donation intervals [18, 20]. After genetic quality control, Sun et al. analyzed 2,994 proteins in 3,301 individuals from European with approximately 10.5 million SNPs (available from the IEU GWAS database: https://gwas.mrcieu.ac.uk/). The SNPs that were identified to be associated with plasma ICAM-4 levels at the genome-wide significance level (*P* < 5.0 × 10^− 8^) and were not in linkage disequilibrium (LD) with other SNPs (r^2^ < 0.1 within a clumping window of 500 kb) were selected as genetic instruments for plasma ICAM-4 levels. In addition, if the ICAM-4-associated SNP was not available in the MEGASTROKE dataset, a proxy SNP (r^2^ > 0.8) was selected by default based on a 1000 Genomes European reference panel. Overall, a total of 11 SNPs were selected as the genetic instruments for plasma ICAM-4 in this MR study (Table 1).

**Table 1:** Characteristics of genetic variants proxied for intercellular adhesion molecule 4

SNP	Chr	Position (build 37)	Nearest Gene	EA	OA	EAF	Beta	SE	*P* value
rs113415585	1	25,554,998	*SYF2*	A	G	0.03694	-0.5040	0.0739	9.12 × 10^− 12^
rs149152056^*^	1	25,705,424	*RHCE*	T	C	0.03092	-0.4119	0.0711	6.92 × 10^− 9^
rs2375113	1	25,525,915	*SYF2*	G	A	0.20298	0.2540	0.0301	3.31 × 10^− 17^
rs28608145	1	25,520,341	*SYF2*	G	C	0.97613	-0.6041	0.0875	5.01 × 10^− 12^
rs4649080^*^	1	25,737,116	*RHCE*	T	C	0.08227	-0.4015	0.0484	1.02 × 10^− 16^
rs61774838	1	25,482,196	*IFITM3P7*	A	G	0.13531	-0.2645	0.0381	4.07 × 10^− 12^
rs72660908	1	25,583,610	*RSRP1*	G	C	0.45371	-0.6177	0.0230	4.68 × 10^− 159^
rs72660919	1	25,677,605	*TMEM50A*	A	G	0.16694	0.3113	0.0376	1.35 × 10^− 16^
rs760970	1	25,578,944	*RSRP1*	G	A	0.88987	-0.5808	0.0389	1.86 × 10^− 50^
rs79017607	1	25,519,831	*SYF2*	C	T	0.03393	0.4519	0.0713	2.34 × 10^− 10^
rs79561453	1	25,564,304	*SYF2*	C	G	0.05498	-0.4092	0.0577	1.29 × 10^− 12^

^*^Proxy SNPs (rs150766331 and rs116137570) correlated (r^2^ > 0.8) with SNPs that were not available in the MEGASTROKE dataset

Abbreviations: SNP: single-nucleotide polymorphism; Chr: chromosome; EA: effect allele; OA: other allele; EAF, effect allele frequency; SE: standard error

### Data source for ischemic stroke and its subtypes

In the present study, genetic association data of ischemic stroke and its subtypes were obtained from the previously published GWAS released by the MEGASTROKE project, which was a large-scale international collaboration launched by the International Stroke Genetics Consortium [19]. This dataset was based on a meta-analysis of 29 European-ancestry GWASs with 8 million SNPs, involving 34,217 ischemic stroke cases and 406,111 controls. Among these ischemic stroke cases, 7,193 cases were CES, 4,373 cases were LAS, and 5,386 cases were SVS according to the Trial of Org 10,172 in Acute Stroke Treatment criteria [21].

### Statistical analysis

A two-sample MR analysis was performed to estimate the associations of genetically determined plasma ICAM-4 with the risks of ischemic stroke and its subtypes using summarized data of the SNP-ICAM-4 and SNP-ischemic stroke. We used F statistics with the formula F = ($\frac{N-K-1}{K}$) ($\frac{{R}^{2}}{1-{R}^{2}}$) to measure the strength of instrumental variables, where *R*^2^ was the proportion of variation in plasma ICAM-4 levels explained by the instrumental SNPs, N was the sample size, and K was the number of SNPs used as genetic instruments of plasma ICAM-4 [22]. In general, a mean F statistic greater than 10 ensured negligible bias from weak instruments [23]. In addition, an online web tool named mRnd (https://shiny.cnsgenomics.com/mRnd/) was used to calculate the statistical power of the present MR study [24].

Conventionally, under the causal null hypothesis that all instrumental variables were valid and pleiotropic balanced, the inverse-variance weighted (IVW) method with the greatest statistical power was the most efficient way to combine them into a single variant-specific causal estimate and it generally was used as the primary analysis method in MR study [25–27]. Therefore, we used the IVW method as the main analysis to evaluate the associations of genetically determined plasma ICAM-4 with the risks of ischemic stroke and its subtypes in the present MR study. The heterogeneity among genetic instruments was assessed via Cochran’s Q statistic, which was a weighted sum of the squared distances of the variant-specific estimates from the overall IVW estimate [27, 28]. A large value of the Q statistic meant that the variant-specific ratio estimates differ by more than expected due to chance alone and significant *P*-value with Q-statistic was usually set to 0.05 [28, 29]. To avoid possible causal estimates bias that might be caused by fitting different models, we conducted the random-effect IVW model and fixed-effect IVW model to further explore the association between ICAM-4 and ischemic stroke [27, 30].

In the sensitivity analyses, we employed various MR methods (the penalized IVW, [31] maximum likelihood, [32] MR-Robust Adjusted Profile Score [MR-RAPS], [33] and MR-Egger regression [34]) to assess the robustness of our findings. The penalized IVW penalized the weights of SNPs with pleiotropy [31]. The maximum likelihood method was applied to provide relatively reliable estimates in the presence of measurement error in the SNP-exposure effect [32]. The MR-RAPS analysis could solve the bias of horizontal pleiotropy and weak instruments [33]. The MR-Egger regression method could evaluate the average pleiotropic effects across all SNPs via the intercept term [34].

Results were presented as odds ratios (ORs) with 95% confidence intervals (CIs). A Bonferroni-corrected significance level of 2-sided *P* < 0.0125 (0.05/4 [ischemic stroke and 3 ischemic stroke subtypes]) was considered as the statistically significant evidence for a causal association. All analyses were performed using R software (version 4.1.1; R Development Core Team) with R packages named gtx, TwoSampleMR, and MendelianRandomization.

## Results

A total of 11 SNPs were used as genetic instruments for plasma ICAM-4 levels in the present study, and the details of genetic instruments were provided in Table 1. 11 genetic instruments identified together explained 34.90% variances of plasma ICAM-4 levels, and the statistical power in this MR study ranged from 97 to 100% (Table S1). The F-statistics of instrumental variables was 160.60, indicating that there was no weak instrument bias (Table S1).

### Causal effects of ICAM-4 levels on ischemic stroke and its subtypes

**Fig. 2:**
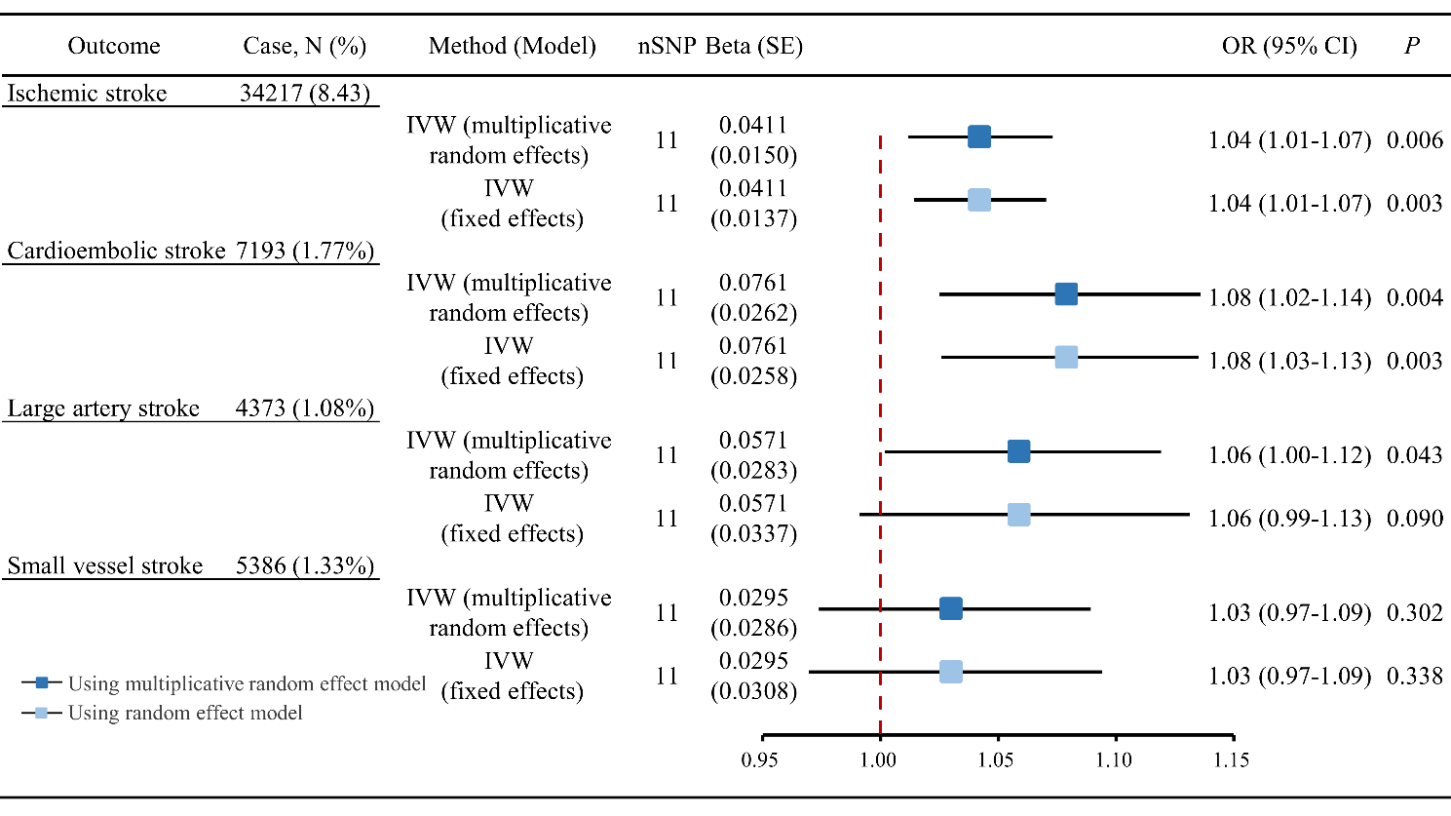
Main analysis for associations of genetically determined ICAM-4 levels with ischemic stroke and its subtypes Abbreviations: ICAM-4, intercellular adhesion molecule 4; IVW, inverse-variance weighted; SNP, single-nucleotide polymorphism; SE: standard error; OR, odds ratio; 95% CI, 95% confidence interval

**Fig. 3:**
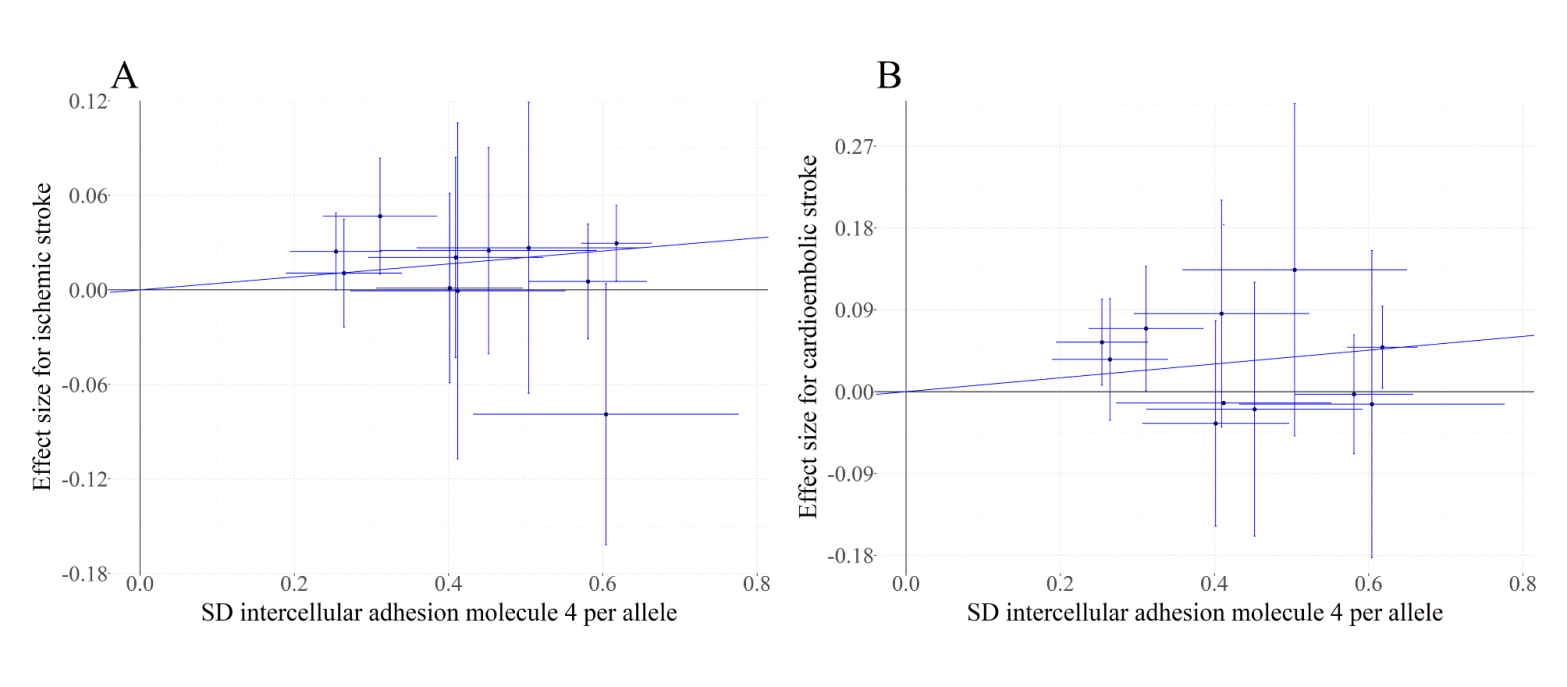
**Associations between genetic instruments of ICAM-4 and the risks of ischemic stroke and cardioembolic stroke**. The line indicates the estimate for the associations of intercellular adhesion molecule 4 (ICAM-4) levels with ischemic stroke and cardioembolic stroke using inverse-variance weighted method. Circles indicate associations of each genetic variant related to ICAM-4 levels with the risks of ischemic stroke and cardioembolic stroke. Genetic error bars indicate 95% confidence intervals A, ischemic stroke; B, cardioembolic stroke

The results from the Cochran’s Q test (Table S2) contained the Q statistic, degrees of freedom, and Q statistic associated *P*-value, and we observed no evidence of heterogeneity between genetic variants (all *P* > 0.05). As illustrated in Fig. 2, the IVW analysis with multiplicative random effects model demonstrated that genetically determined ICAM-4 levels were positively associated with the risks of ischemic stroke (OR per SD [standard deviation] increase: 1.04; 95% CI: 1.01–1.07; *P* = 0.006) and CES (OR per SD increase: 1.08; 95% CI: 1.02–1.14; *P* = 0.004). In contrast, there was no significant association of genetically determined ICAM-4 with LAS (OR per SD increase: 1.06; 95% CI: 1.00-1.12; *P* = 0.043) and SVS (OR per SD increase: 1.03; 95% CI: 0.97–1.09; *P* = 0.302). In the IVW analysis fitting fixed effects model, genetically determined high ICAM-4 levels were associated with increased risks of ischemic stroke (OR per SD increase: 1.04; 95% CI: 1.01–1.07; *P* = 0.003) and CES (OR per SD increase: 1.08; 95% CI: 1.03–1.13; *P* = 0.003) but not with LAS (OR per SD increase: 1.06; 95% CI: 0.99–1.13; *P* = 0.090) and SVS (OR per SD increase: 1.03; 95% CI: 0.97–1.09; *P* = 0.338). Associations between each instrumental variant for ICAM-4 levels and the risks of ischemic stroke and CES were presented in Fig. 3.

### Sensitivity analyses

We conducted sensitivity analyses with a series of MR methods to assess the robustness of our findings (Table 2). Genetically determined ICAM-4 was positively associated with the risk of ischemic stroke in the sensitivity analyses with the penalized IVW method (OR per SD increase:1.04; 95% CI:1.01–1.07; *P* = 0.006), maximum likelihood method (OR per SD increase: 1.04; 95% CI:1.01–1.07; *P* = 0.003), and MR-RAPS method (OR per SD increase: 1.04; 95% CI:1.01–1.07; *P* = 0.003). Similarly, the penalized IVW analysis (OR per SD increase: 1.08; 95% CI:1.02–1.14; *P* = 0.004), maximum likelihood analysis (OR per SD increase: 1.08; 95% CI:1.03–1.14; *P* = 0.003), and MR-RAPS analysis (OR per SD increase: 1.08; 95% CI:1.03–1.14; *P* = 0.003) showed that genetically determined high ICAM-4 levels were associated with an increased risk of CES. In addition, MR-Egger regression suggested that there was no directional pleiotropy for these associations (all *P* > 0.05; Table 2).

**Table 2:** Sensitivity analyses for associations of genetically determined ICAM-4 levels with ischemic stroke and its subtypes

Outcome	Parameter	OR (95% CI)	*P* value
**Ischemic stroke**			
Penalized IVW	OR	1.04 (1.01–1.07)	0.006
Maximum Likelihood	OR	1.04 (1.01–1.07)	0.003
MR-RAPS	OR	1.04 (1.01–1.07)	0.003
MR-Egger	OR	0.98 (0.91–1.06)	0.657
	Odds (intercept)	1.03 (0.99–1.07)	0.118
**Cardioembolic stroke**			
Penalized IVW	OR	1.08 (1.02–1.14)	0.004
Maximum Likelihood	OR	1.08 (1.03–1.14)	0.003
MR-RAPS	OR	1.08 (1.03–1.14)	0.003
MR-Egger	OR	0.95 (0.82–1.10)	0.484
	Odds (intercept)	1.06 (1.00-1.14)	0.066
**Large artery stroke**			
Penalized IVW	OR	1.06 (0.99–1.13)	0.090
Maximum Likelihood	OR	1.06 (0.99–1.13)	0.088
MR-RAPS	OR	1.06 (0.99–1.13)	0.091
MR-Egger	OR	0.89 (0.74–1.08)	0.228
	Odds (intercept)	1.09 (0.90–1.05)	0.055
**Small vessel stroke**			
Penalized IVW	OR	1.03 (0.97–1.09)	0.338
Maximum Likelihood	OR	1.03 (0.97–1.09)	0.342
MR-RAPS	OR	1.03 (0.97–1.09)	0.339
MR-Egger	OR	1.10 (0.92–1.31)	0.300
	Odds (intercept)	0.97 (0.90–1.05)	0.452

Abbreviations: ICAM-4, intercellular adhesion molecule 4; OR, odds ratio; 95% CI, 95% confidence interval; IVW, inverse-variance weighted; MR-RAPS, mendelian randomization robust adjusted profile score

## Discussion

To the best of our knowledge, this is the first study with sufficient statistical power to investigate the associations of genetically determined ICAM-4 levels with the risks of ischemic stroke and its subtypes. In this MR study with 446,696 European participants, we found that genetically determined high ICAM-4 levels were associated with increased risks of ischemic stroke and CES, but not LAS or SVS. Sensitivity analyses using a series of MR methods further confirmed these identified associations.

As the key molecules involved in progression of the ischemia, the upregulation of ICAMs was able to enhance leukocyte-endothelial cell interactions and induce neutrophils infiltrate into damaged brain tissue, thereby aggravating the damage of blood-brain barrier [10]. ICAM-4, an important member of the ICAMs, was well-established to be implicated in hemostasis and thrombosis [6, 7]. Data from animal studies had revealed that ICAM-4 contributed to the adhesion of endothelial cells, thrombosis, and vaso-occlusion through interacting with activated platelets and leukocytes [35, 36]. Vivian et al. found that ICAM-4 could induce massive erythrocytes incorporation into thrombus and activate platelets, while blockading ICAM-4 could cause a reduction of fibrin and thrombus [8]. Therefore, it has been suggested that ICAM-4 may be associated with the development of thrombosis and ischemic stroke, but the population-based evidence is limited so far.

We conducted a systematical MR study to investigate the associations of genetically determined ICAM-4 levels and the risks of ischemic stroke and its subtypes. In this MR study, genetically determined high ICAM-4 levels were observed to be associated with the increased risk of ischemic stroke, suggesting that it might be a promising predictive marker for ischemic stroke. Besides, in the analysis of ischemic stroke subtypes, we found that there were possible mechanism-specific detrimental effects of genetically determined ICAM-4 on CES but not LAS or SVS. Given that ischemic stroke subtypes might differ for the genetic pathophysiological mechanisms, [37] CES had a higher inflammatory environment, macrophage, and platelet content in thrombus than the other stroke subtypes [38, 39]. Therefore, we speculated that elevated ICAM-4 levels were significantly associated with increased risks of ischemic stroke and CES via mediating aggregation and abnormal adhesion of inflammatory cells, macrophages, and platelets. Further studies are warranted to explore the detailed mechanism underlying the association of ICAM-4 with ischemic stroke and CES.

Our findings had significant public health and clinical implications. The present MR study was the first to provide population-based evidence for the associations of ICAM-4 levels with the risks of ischemic stroke and its subtypes from a genetic point of view. Collectively, our study showed that elevated ICAM-4 levels increased the risks of ischemic stroke and CES, suggesting that ICAM-4 might be acted as a promising biomarker to identify high-risk individuals for active monitoring and early intervention of ischemic stroke. In addition, it was of clinical interest to explore whether targeting ICAM-4 or its downstream effectors could reduce the risks of ischemic stroke, especially CES.

The present study had several methodological strengths. First, MR design followed the genetic rule that parental alleles were randomly assigned to offspring and possessed reasonable causal order [11, 12]. Therefore, the implementation of MR approach in this study diminished the interference of confounding factors and reverse causation on the results, which might be more convincing than observational studies [11, 12]. In addition, we used the most comprehensive and the largest available GWASs about ICAM-4 levels, ischemic stroke, and its subtypes, [18, 19] which enabled us to provide a valid appraisal of the associations with the high statistical power. Finally, the significant associations observed in this MR study were subjected to multiple corrected and a series of sensitivity analyses further confirmed our findings.

Our study had several limitations that needed to be interpreted. Firstly, MR analysis might be influenced by instrument bias and potential pleiotropy. However, the F-statistic for the genetic instruments in the present study was greater than 10, suggesting that there was no weak instrument bias. Furthermore, the MR-Egger regression suggested no directional pleiotropy for identified associations in this MR study. Secondly, the present study estimated the lifetime effect of plasma ICAM-4 in the risks of ischemic stroke and its subtypes, so the results should not be directly extrapolated to assess the effect of any potential clinical intervention targeting ICAM-4. Finally, the summary GWAS data we used merely concerned European individuals, so we should cautiously utilize our conclusion in racially and ethnically diverse populations. However, this restriction decreased the possibility of spurious associations due to population stratification bias. Further studies are needed to confirm our findings among individuals of non-European ancestry.

## Conclusions

We found positive associations of genetically determined high plasma ICAM-4 levels with the risks of ischemic stroke and CES. Further studies are needed to verify our findings and explore the detailed mechanism underlying the detrimental effects of ICAM-4 on the risk of ischemic stroke.

## Electronic supplementary material

Below is the link to the electronic supplementary material.

Supplementary Material 1

## Data Availability

The public summary statistic data for ICAM-4, ischemic stroke and ischemic stroke subtypes during the current study are available from the IEU GWAS database (https://gwas.mrcieu.ac.uk/). Statistical code analysed during the current study are available from the corresponding author on reasonable request. (email: Zhengjin22163@fudan.edu.cn or zbzhu@suda.edu.cn).

## List of abbreviations

ICAM-4: Intercellular adhesion molecule 4
MR: Mendelian randomization
GWAS: Genome-wide association studies
OR: Odds ratio
SD: Standard deviation
CI: Confidence interval
CES: Cardioembolic stroke
LAS: Large artery stroke
SVS: Small vessel stroke
STROBE-MR: Strengthening the Reporting of Observational Studies in Epidemiology using Mendelian Randomization
SNPs: Single-nucleotide polymorphisms
MEGASTROKE: Multi-ancestry Genome-Wide Association Study launched by the International Stroke Genetics Consortium
LD: Linkage disequilibrium
IVW: Inverse-variance weighted
MR-RAPS: Mendelian randomization-Robust Adjusted Profile Score
