# Supplementary material for: Intercellular adhesion molecule 4 and ischemic stroke: a two-sample Mendelian randomization study
Source: Thromb J. 2023 Apr 11;21:40. doi: 10.1186/s12959-023-00485-4 (PMC10091569; doi:10.1186/s12959-023-00485-4)
Supplement: Supplementary file 1 — Supplementary Material 1 [file 12959_2023_485_MOESM1_ESM.docx]

**SUPPLEMENTAL MATERIAL**

**Table S1.** F-statistic estimates of genetic instruments and the statistical power (%) in the mendelian randomization study

**Table S2.** Results of heterogeneity test in mendelian randomization analysis

**Table S1.** **F-statistic estimates of genetic instruments and** **the statistical power (%) in the mendelian randomization study**

| Outcome | Intercellular adhesion molecule 4 (F^a^=160.60) | |
| --- | --- | --- |
|  | R^2^ | Power^b^ |
| Ischemic stroke | 0.349 | 100.00 |
| Cardioembolic stroke | 0.349 | 100.00 |
| Large artery stroke | 0.349 | 97.00 |
| Small vessel stroke | 0.349 | 99.00 |

^a^ F-statistics that quantified the strength of the selected instrumental variables were done with the formula of F = ($\frac{N-K-1}{K}$) ($\frac{R^{2}}{1-R^{2}}$), where R^2^ was the proportion of variation in intercellular adhesion molecule 4 levels explained by the SNPs, N was the sample size, and K was the number of SNPs in genetically proxied intercellular adhesion molecule 4 levels.

^b^ Power was calculated using the previously described online method (https://shiny.cnsgenomics.com/mRnd/), which represented statistical power to detect an odds ratio of 1.10 per standard deviation increment in intercellular adhesion molecule 4 at a 5% false positive rate.

**Table S2. Results of heterogeneity test in Mendelian randomization analysis**

| Outcome | Q^a^ value | Degrees of freedom | *P*^b^ value |
| --- | --- | --- | --- |
| Ischemic stroke | 12.07 | 10 | 0.28 |
| Cardioembolic stroke | 10.37 | 10 | 0.41 |
| Large artery stroke | 7.03 | 10 | 0.73 |
| Small vessel stroke | 8.61 | 10 | 0.57 |

^a^ Heterogeneity between the variant-specific causal estimates could be measured using Cochran’s Q statistic with the formula$Q=\sum_{j} \mathrm{se}{(\hat{\theta}_{j})}^{-2} {(\hat{\theta}_{IVW-}\hat{\theta}_{j})}^{-2}$, which was a weighted sum of the squared distances of the variant-specific estimates from the overall IVW estimate.

^b^ Following common practice, one may label a collection of differences between the variant-specific ratio estimates as “heterogeneous” when the corresponding Q-statistic p-value is below a nominal cut-off (usually 0.05).
